# Horizon scanning in Brazil: outputs and repercussions

**DOI:** 10.11606/S1518-8787.2019053001439

**Published:** 2019-11-21

**Authors:** Pollyanna Teresa Cirilo Gomes, Verónica Elizabeth Mata, Thais Conceição Borges, Dayani Galato

**Affiliations:** I Ministério da Saúde. Departamento de Gestão e Incorporação de Tecnologias em Saúde. Brasília, Distrito Federal (DF), Brasil; II Universidade de Brasília. Faculdade de Ceilândia. Programa de Pós-Graduação em Ciências e Tecnologias da Saúde. Distrito Federal (DF), Brasil

**Keywords:** Technology Assessment, Biomedical, Technology Control, Biomedical, Health Sciences, Technology, and Innovation Management, Policies and Cooperation in Science, Technology and Innovation, Evidence-Informed Policy

## Abstract

**OBJECTIVE:**

To describe the four types of horizon scanning (HS) outputs developed by the National Committee for Health Technology Incorporation (CONITEC) and show their main repercussions on the decision-making processes of the Brazilian Ministry of Health (MH).

**METHODS:**

Descriptive study based on participant observation and document analysis of HS outputs (internal reports, alert reports, briefs and sections for CONITEC recommendation reports) developed between January 2014 and July 2018.

**RESULTS:**

Fifteen internal reports, six alert reports, two briefs and 57 HS sections were produced. Each output has a specific structure according to its purpose. The methodological approach adopted for developing HS outputs in Brazil is described by EuroScan International Network. The outputs had institutional and international repercussions. The activities resulted in the inclusion of HS as a tool for reducing health lawsuits in the legal framework of the MH. One of the internal reports on a high-cost drug not approved in Brazil for a rare disease was requested by the Health Technology Assessments Network for the Americas (RedETSA), showing the international relevance of the outputs. The HS sections in recommendation reports influenced discussions about incorporating technologies into the Unified Health System.

**CONCLUSIONS:**

The developed outputs have purposes ranging from helping build arguments for defense of the MH in cases of health judicialization to inform decision-making processes. In addition, HS sections in recommendation reports have grown in importance recently. CONITEC’s HS system has been structured, and its role as a tool to inform health managers has shown to be been relevant.

## INTRODUCTION

In the last decades, the health field has been marked by the profusion of new technologies, not always safe, effective or with clinical superiority over those already available. Demand for health services and technologies has increased exponentially, with consequences for the allocation of human and financial resources, as well as the logistics for the implementation of health services^1^.

Facing the challenge of defining which technologies will be enabled by health systems, the use of health technology assessment (HTA) is increasing in support of decision-making processes^2^. The incorporation, exclusion and alteration of technologies offered by the Unified Health System (SUS) are carried out with the advice of the National Committee for Health Technology Incorporation (CONITEC), with the application of the HTA^3^^,^^4^^,^^5^.

One of the HTA phases is horizon scanning (HS), which is the systematic identification of new and emerging technologies with the potential to impact health, health systems and/or society, with the purpose of timely informing decision-makers^6^. New technologies are those in the launching phase or in the early stages of diffusion of use in the health care system. Emerging technologies are in phases 2 or 3 of clinical research or in the pre-market phase^7^.

Several countries adopt HS as an approach to prepare their health systems for such technologies^8^^–^^12^. Discussions about the organization of a Brazilian HS system started more than 10 years ago through the Brazilian Health Technology Assessment Network (REBRATS)^13^^,^^14^.

Currently, the legal attribution of HS activities at federal level belongs to CONITEC^3^. CONITEC’s HS system is part of the International Information Network on New and Emerging Health Technologies (EuroScan), the largest collaboration network on new and emerging technologies. SUS is the main client of CONITEC’s system^15^^,^^16^.

In response to different information needs, CONITEC’s HS system has developed internal reports, alert reports, briefs and HS sections in the committee’s recommendation reports. The purpose of this study was to describe the main characteristics of these outputs and show the main repercussions generated by HS.

## METHODS

Descriptive study, based on participant observation and documentary analysis of HS outputs prepared under CONITEC, between January 2014 and July 2018. Data collection was performed by searching the CONITEC website^17^. HS internal reports with restricted access were obtained from documentary research after formal authorization from the Ministry of Health (MH).

The publication “A toolkit for the identification and assessment of new and emerging health technologies”^7^ was used as a theoretical framework for the description of (i) internal reports, (ii) alert reports, (iii) briefs and (iv) HS sections in CONITEC recommendation reports. The resulting implications of HS outputs were shown by describing examples of the repercussions of the information in the MH and in the CONITEC plenary decision-making process.

## RESULTS

The outputs were designed by applying the steps of the EuroScan toolkit^7^. The technologies addressed in the outputs were indicated by the information requester (internal reports) or identified by searches in the clinical trial registry database ClinicalTrials.gov; on the websites of health regulatory agencies in Brazil (Brazilian Health Regulatory Agency – ANVISA), Europe (European Medicines Agency – EMA) and the United States of America (Food and Drug Administration – FDA); in addition to EuroScan and Cortellis™ databases. Table 1 shows the characterization of the outputs.

**Table 1:** Characterization of the outputs developed by CONITEC’s horizon scanning system during the analyzed period and the adopted steps, according to the EuroScan toolkit^7^.

	Internal Reports	Alert reports	Briefs	HS Sections in CONITEC Recommendation Reports
Target audience	MH managers	Society^18^	MH health professionals, academy and managers	CONITEC plenary
Purpose	To respond the applicant’s specific questions (answers to court demands)	To disseminate information on new and emerging technologies and warn against the possibility of improper diffusion of these technologies		To support CONITEC Plenary discussions and prepare health care system for new and emerging technologies
Format	Printed	Digital		Digital (in recommendation reports) and oral (in CONITEC meetings)
System scope	Medicines, diagnostic methods, software		Medicines and medical devices	Medicines
Time horizon	Technologies from phase 2 of clinical research; or without licensing with ANVISA, EMA or FDA; or newly approved by these agencies	Technologies from phase 3 of clinical research; or without licensing with ANVISA, EMA or FDA; or newly registered at these agencies	Technologies from phase 2 of clinical research; or without licensing with ANVISA, EMA or FDA; or newly approved by these agencies	Technologies from phase 3 of clinical research; or without licensing with ANVISA, EMA or FDA; or newly registered at these agencies
Identification	Passive process			
Filtering and prioritization	Performed internally. In case of requesting information about a specific technology, filtering and prioritization were not performed	Performed internally. Application of the criteria: MH spending on technology due to judicialization; burden of disease; availability of therapeutic options for the clinical condition in SUS; relevance of topic to MH policymakers	Performed internally. Application of the criteria: technologies from phase 2 of clinical research and without licensing for the therapeutic indication in ANVISA or recently registered in the country	Performed internally. Application of the criteria: technologies from phase 3 of clinical research and without licensing for the therapeutic indication in ANVISA or recently registered in the country
Evaluation	The information provided depended on the applicant’s question. No predefined template was established	Predefined template^18^ with information on: analyzed technology, regulatory situation in Brazil and worldwide, clinical research data, existence of Brazilian Clinical Guidelines published for the disease, scientific evidence of effectiveness and safety, technology impact predictions^18^	No predefined template was established. Information related to technology(ies), patient, scientific evidence of efficacy and safety, and impact prediction on patient care and health services were addressed	Predefined template with topics: active principle, mechanism of action, clinical trial status, regulatory data (designation or approval of use as an orphan drug and licensing by ANVISA, EMA and FDA). Efficacy and safety data were presented orally to the CONITEC plenary
Type of evaluation	Rapid, brief or in-depth	Brief	In-depth	Rapid
Dissemination	Restricted circulation to the information requester	CONITEC website and mailing list; wide circulation		CONITEC Website
Peer review	Internal and external review with expert involvement			Internal review, but subject to external review through public consultations to which reports are submitted

HS: horizon scanning; CONITEC: National Committee for Health Technology Incorporation; MH: Ministry of Health; ANVISA: Brazilian Health Regulatory Agency; EMA: European Medicines Agency; FDA: Food and Drug Administration; SUS: Unified Health System

The recipients of output of the study period were MH managers, CONITEC plenary, and society. The outputs were prepared by an internal team of three people with 0.4 full-time equivalent HS workload (1.0 full-time equivalent corresponds to a weekly workload of 40-hours). Some of the outputs were designed by external partners of health technology assessment centers at two hospitals and one university, corresponding to 0.2 full-time equivalents. The outputs comprised four categories of health technologies, totaling 80 documents (Table 2).

**Table 2:** Number of horizon scanning outputs of the study period as to the health technology categories evaluated.

Type of output	Quantity per technology type addressed
Internal report	(12) Medicine (2) Diagnostics (1) Software
Alert report	(5) Medicine (1) Medical device
Brief	(1) Medicine (1) Medical device
HS section in recommendation reports	(57) Medicine
	n = 80

HS: horizon scanning

**Figure 1:**
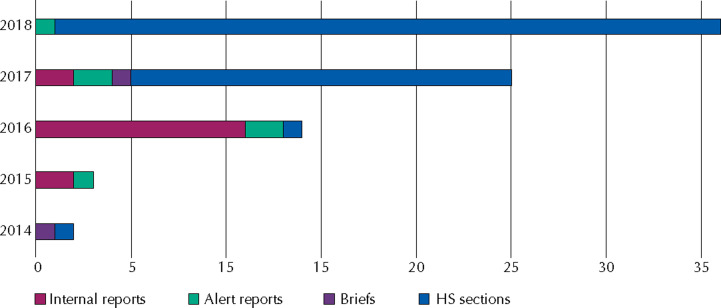
Number of technology horizon monitoring outputs prepared per year evaluated in the study.

Between 2015 and 2016, internal reports predominated (n = 13). Between 2016 and 2018, HS sections in CONITEC recommendation reports were the most frequent output (Figure 1).

The most frequent themes were related to rare diseases (n = 21), rheumatology (n = 10), neurology (n = 9), and oncology (n = 9). The other outputs were related to hematology, pneumology, cardiovascular system, infectology, endocrinology and others.

### Internal reports

Internal reports aimed to support the defense of the MH in cases of drugs required by court; to assist in the definition of medicines to establish Partnerships for Productive Development (PDP); to provide information to patients, MH managers and policy makers; and support the development of Brazilian Clinical Guidelines (PCDT)^19^ (Table 3).

**Table 3:** Topics covered in internal horizon scanning reports and purposes.

Topic	Purpose
Beta idursulfase for mucopolysaccharidosis type 2 (Hunter syndrome)	Judicial defense
Mipomersen for homozygous familial hypercholesterolaemia	
Eculizumab for paroxysmal nocturnal hemoglobinuria and atypical hemolytic uremic syndrome	
Metreleptin for Berardinelli-Seip syndrome	
Pompe disease	Elaboration of PCDT
Fabry’s disease	
Mucopolysaccharidoses type 1 and 2	
Medicines for familial amyloidotic polyneuropathy associated with transthyretin	
Watson robot	To inform MH managers
Rapid diagnostic method of bacterial meningitis	
Diagnostic method of colorectal cancer by stool DNA	
Medicines for chronic hepatitis C	
Severe asthma medications	
Recombinant factors VIII for Haemophilia A	Research and Development (R&D)
Medicines for Amyotrophic Lateral Sclerosis	Preparation for patients and MH managers meeting

PCDT: Brazilian clinical guidelines; MH: Ministry of Health

Two types of internal reports were produced, short and extensive^16^. The first ones, with four to six pages, covered a single technology. This approach, among the types of outputs developed by the HS system, was the most appropriate for delivering timely information on new and emerging medicines to managers, given the short time available for the preparation of MH judicial defenses (Table 3). Internal reports of the second type, the extensive ones, were in-depth reviews of one or several technologies for a given clinical indication, being the useful modality to support the development of clinical guidelines (Table 1).

Internal reports had restricted circulation. However, those related to familial amyloid polyneuropathy and homozygous familial hypercholesterolaemia were pertinent to other audiences. Thus, they were adapted to the brief and alert report formats, respectively.

One of the internal reports that exemplifies implications of HS activities in MH is the metreleptin report. In 2015, lawsuits were submitted to the MH requiring the drug for patients with Berardinelli-Seip syndrome, a rare disease consisting of congenital generalized lipodystrophy^20^. The drug, which has not been registered in Brazil, is a recombinant analogue of the human hormone leptin and had been approved by FDA in February 2014 for that clinical indication^21^. Each ampule of metreleptin costs about US$ 1,766.40^22^, with an estimated spending of over $ 4 million per year to treat patients. At that time, efficacy and safety data on the drug were scarce, and there was no HS study on the subject in the EuroScan database.

The HS internal report^23^ contained information on the disease, epidemiological data from Brazil and around the world, the description of the drug, the estimated costs of treatment with metreleptin, and evidence on efficacy and safety, as well as regulatory and clinical research situations for therapeutic indication. Despite the weak evidence in favor of the drug use to treat the disease, court decisions were favorable to its costing.

Subsequently, the HS report^23^ was shared with Argentina. The request for information was made through the Health Technology Assessments Network for the Americas (RedETSA). In Argentina, the drug was being requested for compassionate use of patients with the disease, which could result in high expenses, as in Brazil.

### Alert reports

The purpose of alert reports was to predict the impact of new and emerging legally demanded technologies, as well as those that could be brought to court in the future. The recipients of the information were judges, patients, health professionals and managers, which was reflected on the use of simple language and brief extension. The alert reports included a technology for a therapeutic indication^18^.

Six alerts were produced in the period: ledispavir associated with sofosbuvir for chronic Hepatitis C genotype 1, eliglustate tartrate for type 1 Gaucher disease, mipomersen for homozygous familial hypercholesterolemia, ivacaftor for cystic fibrosis, medical device for severe mitral regurgitation in patients with high surgical risk, and aducanumab for Alzheimer’s disease (Table 1).

### Briefs

Briefs were intended to show potential new and emerging technologies for a health condition, addressing various technologies (Table 1). Two reports were produced during the study period: “Bioabsorbable stents in percutaneous coronary intervention” and “Drugs under development for treating familial amyloid polyneuropathy associated with transthyretin.” These briefs covered two categories of technologies, medical devices and medicines (Figure 1), with the main target audience being health professionals and the academy.

### HS Sections in Recommendation Reports

CONITEC’s recommendation reports are official MH documents that include scientific evidence, economic evaluation, and budget impact assessment of health technologies submitted for analysis for incorporation into SUS^5^. HS sections had aimed to present the drugs that could potentially compete with the one being analyzed for incorporation into SUS, either by new route of administration or by representing a new therapeutic class, for example, to support the discussions of CONITEC^5^.

Pilot analyses of the technological landscape of drugs in clinical development for multiple sclerosis were performed during the evaluation of the demands of incorporation of fingolimod and teriflunomide in 2014 and 2016, respectively. The HS findings were shown to the CONITEC plenary, influencing the recommendation issued. Due to HS’s role in decision-making, this output was systematically made for each drug under review by the commission^24^ as of 2017.

Between 2017 and July 2018, 58 HS sections were prepared, an average of three sections per month. The total of 153 new and emerging technologies were prospected. The most frequent health topics were rare diseases (23%), oncology (19%) and neurology (17%). In addition to being included in the recommendation report, the information was presented orally to the plenary (Table 1) and impacted in discussions and decision-making processes.

One of these repercussions occurred in the context of the analysis to incorporate adalimumab, etanercept, infliximab, secukinumab and ustekinumab for moderate to severe psoriasis^25^. HS appointed 13 drugs for that clinical indication, and efficacy data for guselkumab, ixekizumab and brodalumab suggested superiority over adalimumab, etanecerpt, infliximab, and ustekinumab.

In addition, three emerging drugs (pliclidenoson, tofacitinib and voclosporin) were being developed for oral administration. On the other hand, all drugs under analysis for incorporation were administered subcutaneously or intravenously, indicating a potential positive repercussion of these technologies on patients’ therapeutic compliance, if they were registered in the country.

The scenario of potentially more effective drugs with more convenience for the patient in the near future resulted in the intensification of the CONITEC plenary debate on the preliminary recommendation not to incorporate three of the drugs under consideration.

In 2017, institutional recognition of the key role of CONITEC’s HS system in providing strategic information on new and emerging technologies to the MH resulted in the legal inclusion of horizon scanning as an activity of the commission’s advisory department as a tool to reduce health lawsuits^26^.

## DISCUSSION

This study describes HS outputs developed by the Brazilian MH in order to support the health technologies assessment in the context of one of the largest health systems in the world. The results show that the EuroScan toolkit^7^ has been applicable to the Brazilian HS system. The increase in the number of outputs produced per year shows that, over time, the potential of HS as a tool for collecting, synthesizing and presenting information on new and emerging technologies has been adopted in the decision-making processes in MH.

We found that, although within the same HS system, the HS steps were not uniquely employed to build different outputs to provide information for specific audiences. The study points out a differential among Brazilian HS outputs: their close relation with the judicialization of health, in which technologies are required through the judiciary. This access mechanism has been a gateway for high cost drugs with great potential for inadequate diffusion and irrational use, especially of technologies not approved by ANVISA^27^. According to Douw et al.^28^ (2003), HS systems are intended to help streamline the adoption and diffusion processes of new technologies. The potential positive effect of HTA in the preparation of defenses in cases of health judicialization has been pointed out^29^.

The HS has informed the Brazilian judicial system through the arguments shown in the defense pieces of the MH in court and, secondarily, through alert reports and briefs. This is caused because these HS outputs appear among search results for technologies not yet approved by ANVISA on tools such as Google®, as they are disseminated through the CONITEC website.

The sharing of metreleptin’s internal report with Argentina, case presented in this study, shows that CONITEC’s HS outputs can be used by other countries dealing with judicialization as well as other purposes. The generation of scientific evidence was identified as one of the priority strategies for addressing health judicialization in Latin America and the Caribbean^30^, showing the potential use of information provided by HS outputs for the region.

In the study by Packer et al.^9^ (2015) on the structure, processes and outputs of 15 EuroScan members, 80% of the HS systems studied reported peer review expert involvement. Some of CONITEC’s HS outputs are also peer reviewed. As with most HS systems reported in this study, the data in this investigation indicate that there is more than one group of potential users of the information generated by this system.

As the Swedish agency’s HS reports, Brazilian outputs do not constitute a complete assessment of new or emerging technology but provide managers with early information about them^11^.

Like other EuroScan members, CONITEC’s HS team is small, consisting of three people involved in other activities as well. CONITEC’s HS system is part of a department that operates in health technology assessment activities, not constituting thus a separate institution, as well as other members of the international network^9^.

Literature on the results of HS activities is scarce^9^^,^^32^^,^^33^. The information provided by the HS sections in the recommendation reports has influenced CONITEC’s recommendations. This allows us to state that the system described in this study generates repercussions on decision making for the incorporation of technologies into SUS.

From this perspective, the increase in the number of “HS sections in recommendation reports” outputs in 2017 and 2018 shows their relevance to the MH. In 75% of HS systems, the main purpose of the activities is to support coverage and reimbursement decisions^9^, functions similar to those in the HS sections in the recommendation reports.

An important step of the Brazilian HS system will be to carry out studies focused on specific diseases^31^ and deliver this information to the corresponding thematic areas of the MH, in order to indicate technologies with potential for incorporation into SUS. In the United Kingdom, HS activities provide the National Institute for Health and Care Excellence (NICE) with information on technology incorporation^32^.

Although CONITEC cannot proactively guide the technologies that will be analyzed for incorporation, the internal areas of the MH may demand these evaluations from the commission^4^. Thus, the achievement of the step discussed in the previous paragraph may result in a more proactive profile of activities performed by the CONITEC’s HS system. In this sense, the expectation that the HS system could identify early adopting technologies that need to be evaluated for proper use has been partially met^13^.

Translating briefs and alert reports into English and Spanish and making them available in the EuroScan database and the CONITEC website will allow the use of information by institutions conducting HTA and HS activities worldwide. Other challenges for CONITEC’s HS system will be to show information on drug obsolescence and shortages^12^, as well as to improve the dissemination strategies of the developed outputs.

The construction of the Brazilian HS system has involved the Ministry of Health, academia and other stakeholders. These activities have resulted in the institutional recognition of the methodology as an important HTA phase to inform health managers about the best evidence of new and emerging technologies with the potential to have legal, ethical, organizational, and patient care implications.
