# Supplementary material for: Horizon scanning in Brazil: outputs and repercussions
Source: Rev Saude Publica. 2019 Nov 21;53:111. doi: 10.11606/S1518-8787.2019053001439 (PMC9586449; doi:10.11606/S1518-8787.2019053001439)
Supplement: Translation

# Translation: Monitoramento do horizonte tecnológico no Brasil: produtos e repercussões

**Authors:** Pollyanna Teresa Cirilo Gomes, Verónica Elizabeth Mata, Thais Conceição Borges, Dayani Galato

**Affiliations:** I Ministério da Saúde. Departamento de Gestão e Incorporação de Tecnologias em Saúde. Brasília, Distrito Federal (DF), Brasil; II Universidade de Brasília. Faculdade de Ceilândia. Programa de Pós-Graduação em Ciências e Tecnologias da Saúde. Distrito Federal (DF), Brasil

**Keywords:** Avaliação da Tecnologia Biomédica, Controle da Tecnologia Biomédica, Gestão de Ciência, Tecnologia e Inovação em Saúde, Políticas e Cooperação em Ciência, Tecnologia e Inovação, Política Informada por Evidências

## Abstract

**OBJETIVO:**

Descrever os quatro tipos de produtos de monitoramento do horizonte tecnológico (MHT) elaborados no âmbito da Comissão Nacional de Incorporação de Tecnologias no Sistema Único de Saúde (Conitec) e apresentar suas principais repercussões nos processos de tomada de decisão do Ministério da Saúde brasileiro (MS).

**MÉTODOS:**

Estudo descritivo baseado em observação participante e análise documental dos produtos de MHT (relatórios internos, alertas, informes e seções para os relatórios de recomendação da Conitec) elaborados entre janeiro de 2014 e julho de 2018.

**RESULTADOS:**

Foram produzidos 15 relatórios internos, seis alertas, dois informes e 57 seções de MHT. Os produtos têm formatos diferentes, em especial conforme a finalidade. A abordagem metodológica adotada para o desenvolvimento dos produtos de MHT no Brasil está de acordo com o descrito pela EuroScan International Network. Observaram-se repercussões institucionais e internacional dos produtos. As atividades de MHT resultaram na inclusão do monitoramento de tecnologias novas e emergentes como ferramenta para a redução das ações judiciais em saúde no arcabouço legal do MS. Um dos relatórios internos sobre um medicamento de alto custo sem registro no Brasil para doença rara foi requerido pela Rede de Avaliação de Tecnologias em Saúde das Américas, demonstrando a relevância internacional dos produtos. As seções de MHT nos relatórios de recomendação influenciaram as discussões sobre a incorporação de tecnologias no Sistema Único de Saúde.

**CONCLUSÕES:**

Os produtos desenvolvidos apresentam finalidades que vão desde auxiliar na construção de argumentos para defesa do MS em casos de judicialização da saúde até informar processos para a tomada de decisão. Além disso, nos últimos anos as seções de MHT nos relatórios de recomendação têm apresentado crescente importância. O sistema de MHT vinculado à Conitec tem se estruturado, e seu papel como instrumento para informar gestores de saúde tem sido relevante.

## INTRODUÇÃO

Nas últimas décadas, o campo da saúde tem sido marcado pela profusão de novas tecnologias, nem sempre seguras, eficazes ou com superioridade clínica em relação às já disponíveis. A demanda por serviços e tecnologias em saúde aumentou exponencialmente, com consequências na alocação de recursos humanos e financeiros, assim como na logística de implementação dos serviços sanitários^1^.

Perante o desafio de definir quais tecnologias serão disponibilizadas pelos sistemas de saúde, é crescente o uso da avaliação de tecnologias em saúde (ATS) como apoio aos processos de tomada de decisão^2^. A incorporação, exclusão e alteração das tecnologias ofertadas pelo Sistema Único de Saúde (SUS) são realizadas com o assessoramento da Comissão Nacional de Incorporação de Tecnologias no Sistema Único de Saúde (Conitec), com aplicação da ATS^3^^,^^4^^,^^5^.

Uma das fases da ATS é o monitoramento do horizonte tecnológico (MHT), que é a identificação sistemática de tecnologias novas e emergentes com potencial de impactar a saúde, os sistemas de saúde e/ou a sociedade, com a finalidade de informar os tomadores de decisão em tempo oportuno^6^. As tecnologias novas são aquelas em fase de lançamento ou nos estágios iniciais de difusão de uso no sistema de saúde. Tecnologias emergentes estão nas fases 2 ou 3 de pesquisa clínica ou em fase de pré-lançamento no mercado^7^.

Diversos países adotam o MHT como abordagem para preparar seus sistemas de saúde para o advento de tais tecnologias^8^^–^^12^. As discussões sobre a organização de um sistema de MHT brasileiro iniciaram-se há mais de 10 anos por meio da Rede Brasileira de Avaliação de Tecnologias em Saúde (Rebrats)^13^^,^^14^.

Atualmente, a atribuição legal das atividades de MHT no âmbito federal é da Conitec^3^. O sistema de MHT da Conitec integra a *International Information Network on New and Emerging Health Technologies* (EuroScan), a maior rede de colaboração sobre tecnologias novas e emergentes. O principal cliente do sistema da CONITEC é o SUS^15^^,^^16^.

Em resposta a diferentes necessidades de informações, o sistema de MHT da Conitec desenvolveu relatórios internos, alertas, informes e seções de MHT nos relatórios de recomendação da comissão. O propósito deste trabalho foi descrever as principais características desses produtos e apresentar as principais repercussões geradas pelas informações de MHT.

## MÉTODOS

Estudo descritivo, baseado em observação participante e análise documental dos produtos de MHT elaborados no âmbito da Conitec, entre janeiro de 2014 e julho de 2018. A coleta de dados foi realizada por busca no sítio eletrônico da Conitec^17^. Os relatórios internos de MHT, de acesso restrito, foram obtidos a partir de pesquisa documental após autorização formal do Ministério da Saúde (MS).

A publicação “*A toolkit for the identification and assessment of new and emerging health technologies*”^7^ foi utilizada como referencial teórico para a descrição dos (i) relatórios internos, (ii) alertas, (iii) informes e (iv) seções de MHT nos relatórios de recomendação da Conitec. As implicações resultantes dos produtos de MHT foram apresentadas por descrição de exemplos de repercussões das informações no MS e no processo de tomada de decisão do plenário da Conitec.

## RESULTADOS

Os produtos foram elaborados por meio da aplicação das etapas do manual da EuroScan^7^. As tecnologias abordadas nos produtos foram indicadas pelo demandante da informação (relatórios internos) ou identificadas por buscas na base de registro de ensaios clínicos ClinicalTrials.gov; nos sítios eletrônicos das agências reguladoras sanitárias do Brasil (Agência Nacional de Vigilância Sanitária – Anvisa), Europa (*European Medicines Agency* – EMA) e dos Estados Unidos da América (*Food and Drug Administration* – FDA); além das bases de dados da EuroScan e Cortellis™. A caracterização dos produtos está apresentada na Tabela 1.

**Tabela 1:** Caracterização dos produtos desenvolvidos pelo sistema de monitoramento do horizonte tecnológico da Conitec no período analisado e das etapas adotadas, de acordo com o manual da EuroScan^7^.

	Relatórios internos	Alertas	Informes	Seções de MHT nos relatórios de recomendação da Conitec
Público-alvo	Gestores do MS	Sociedade^18^	Profissionais de saúde, academia e gestores do MS	Plenário da Conitec
Finalidade	Responder a questões específicas do solicitante (respostas a demandas judiciais)	Promover a difusão de informações sobre tecnologias novas e emergentes, além de alertar sobre a possibilidade da difusão inadequada dessas tecnologias		Embasar as discussões do Plenário da Conitec e preparar o sistema de saúde para a entrada de tecnologias novas e emergentes
Formato	Impresso	Eletrônico		Eletrônico (nos relatórios de recomendação) e oral (nas reuniões da Conitec)
Escopo do sistema	Medicamentos, métodos diagnósticos, *software*	Medicamentos e dispositivos médicos		Medicamentos
Horizonte de tempo	Tecnologias a partir da fase 2 de pesquisa clínica; ou sem registro na Anvisa, EMA ou FDA; ou recém-registradas nessas agências	Tecnologias a partir da fase 3 de pesquisa clínica; ou sem registro na Anvisa, EMA ou FDA; ou recém-registradas nessas agências	Tecnologias a partir da fase 2 de pesquisa clínica; ou sem registro na Anvisa, EMA ou FDA; ou recém-registradas nessas agências	Tecnologias a partir da fase 3 de pesquisa clínica; ou sem registro na Anvisa, EMA ou FDA; ou recém-registradas nessas agências
Identificação	Processo passivo			
Filtragem e priorização	Realizadas internamente. Nos casos de solicitação de informações sobre uma tecnologia específica, a filtragem e a priorização não foram realizadas	Realizadas internamente. Aplicação dos critérios: gastos do MS com a tecnologia devido à judicialização; carga da doença; disponibilidade de opções terapêuticas para a condição clínica no SUS; relevância do tópico para os formuladores de políticas do MS	Realizadas internamente. Aplicação dos critérios: tecnologias a partir da fase 2 de pesquisa clínica e sem registro para a indicação terapêutica na Anvisa ou registradas recentemente no País	Realizadas internamente. Aplicação dos critérios: tecnologias a partir da fase 3 de pesquisa clínica e sem registro para a indicação terapêutica na Anvisa ou registradas recentemente no País
Avaliação	A informação fornecida dependeu da pergunta do demandante. Não se estabeleceu um modelo pré-definido	Modelo pré-definido^18^ com informações sobre: tecnologia analisada, situação regulatória no Brasil e no mundo, dados de pesquisas clínicas, existência de protocolo clínico e diretrizes terapêuticas publicado para a doença, evidências científicas de eficácia e segurança, estimativas de impacto da tecnologia^18^	Não se estabeleceu um modelo pré-definido. Abordaram-se informações relacionadas à(s) tecnologia(s), paciente, evidências científicas de eficácia e segurança e predição de impacto no cuidado oferecido aos pacientes e nos serviços de saúde	Modelo pré-definido com os tópicos: princípio ativo, mecanismo de ação, status dos ensaios clínicos, dados regulatórios (designação ou aprovação de uso como droga órfã e registro na Anvisa, EMA e FDA). Dados de eficácia e segurança foram apresentados oralmente ao plenário da Conitec
Tipo de avaliação	Rápida, breve ou profunda	Breve	Profunda	Rápida
Disseminação	Circulação restrita ao solicitante	Sítio eletrônico da Conitec e lista de e-mails; circulação ampla		Sítio eletrônico da Conitec
Revisão por pares	Revisão interna e externa com envolvimento de especialistas			Revisão interna, mas é passível de revisão externa por meio das consultas públicas às quais os relatórios são submetidos

MHT: monitoramento do horizonte tecnológico; Conitec: Comissão Nacional de Incorporação de Tecnologias no Sistema Único de Saúde; MS: Ministério da Saúde; Anvisa: Agência Nacional de Vigilância Sanitária; EMA: *European Medicines Agency*; FDA: *Food and Drug Administration*; SUS: Sistema Único de Saúde

Os destinatários das informações dos produtos do período estudado foram gestores do MS, o plenário da Conitec e a sociedade. Os produtos foram preparados por uma equipe interna de três pessoas com carga de trabalho para atividades de MHT de 0,4 equivalentes a tempo integral (1,0 equivalente a tempo integral corresponde a uma pessoa com carga semanal de trabalho de 40 horas). Alguns dos produtos foram elaborados por parceiros externos de núcleos de avaliações de tecnologias em saúde de dois hospitais e uma universidade, correspondendo a 0,2 equivalentes a tempo integral. Os produtos contemplaram quatro categorias de tecnologias em saúde, totalizando 80 documentos (Tabela 2).

**Tabela 2:** Número de produtos de monitoramento do horizonte tecnológico do período estudado com relação às categorias de tecnologias em saúde avaliadas.

Tipo de produto	Quantidade por tipo de tecnologia abordada
Relatório interno	(12) Medicamento (2) Método diagnóstico (1) *Software*
Alerta	(5) Medicamento (1) Dispositivo médico
Informe	(1) Medicamento (1) Dispositivo médico
Seção de MHT nos relatórios de recomendação	(57) Medicamento
	n = 80

MHT: monitoramento do horizonte tecnológico

Entre 2015 e 2016, os relatórios internos predominaram (n = 13). Já entre 2016 e 2018, as seções de MHT nos relatórios de recomendação da Conitec foram o tipo de produto mais frequente (Figura 1).

**Figura 1:** Número de produtos de monitoramento do horizonte tecnológico elaborados por ano avaliado no estudo.

Os temas mais frequentes se relacionaram a doenças raras (n = 21), reumatologia (n = 10), neurologia (n = 9) e oncologia (n = 9). Os demais produtos relacionaram-se à hematologia, à pneumologia, ao sistema cardiovascular, à infectologia, à endocrinologia e outros.

### Relatórios Internos

Os relatórios internos tiveram o objetivo de embasar a defesa do MS em casos de medicamentos requeridos por via judicial; auxiliar a definição de medicamentos para o estabelecimento de parcerias de desenvolvimento produtivo (PDPs); fornecer informações a pacientes, gestores e formuladores de políticas do MS; e dar suporte à elaboração de protocolos clínicos e diretrizes terapêuticas (PCDT)^19^ (Tabela 3).

**Tabela 3:** Tópicos abordados nos relatórios internos de monitoramento do horizonte tecnológico e finalidades.

Tópico	Propósito
Idursulfase beta para mucopolissacaridose tipo 2 (síndrome de Hunter)	Defesa judicial
Mipomersen para hipercolesterolemia familiar homozigótica	
Eculizumabe para hemoglobinúria paroxística noturna e síndrome hemolítico-urêmica atípica	
Metreleptina para síndrome de Berardinelli-Seip	
Doença de Pompe	Elaboração de PCDT
Doença de Fabry	
Mucopolissacaridoses tipo 1 e 2	
Medicamentos para polineuropatia amiloidótica familiar associada à transtirretina	
Robô Watson	Informar gestores do MS
Método diagnóstico rápido de meningites bacterianas	
Método diagnóstico de câncer colorretal por DNA de fezes	
Medicamentos para hepatite C crônica	
Medicamentos para asma grave	
Fatores VIII recombinantes para hemofilia A	Pesquisa e desenvolvimento (P&D)
Medicamentos para esclerose lateral amiotrófica	Preparo para reunião de pacientes e gestores do MS

PCDT: protocolos clínicos e diretrizes terapêuticas; MS: Ministério da Saúde

Foram produzidos dois tipos de relatórios internos, os curtos e os extensos^16^. Os primeiros, de quatro a seis páginas, abordavam uma única tecnologia. Essa abordagem, dentre os tipos de produtos elaborados pelo sistema de MHT, foi a mais adequada para entregar as informações sobre medicamentos novos e emergentes judicializados aos gestores em tempo oportuno, dado o curto prazo disponível para o preparo das defesas judiciais do MS (Tabela 3). Os relatórios internos do segundo tipo, os extensos, constituíram-se em revisões aprofundadas acerca de uma ou de várias tecnologias para uma determinada indicação clínica, sendo a modalidade útil para subsidiar a elaboração de guias de prática clínica (Tabela 1).

Os relatórios internos tiveram circulação restrita. Contudo, aqueles relacionados à polineuropatia amiloidótica familiar associada à transtirretina e à hipercolesterolemia familiar homozigótica mostraram-se pertinentes a outros públicos. Assim, foram adaptados para os formatos de informe e alerta, respectivamente.

Um dos relatórios internos que exemplifica implicações das atividades de MHT no MS é o relatório da metreleptina. No ano de 2015, foram encaminhadas ações judiciais ao MS que requeriam o medicamento para pacientes com a Síndrome de Berardinelli-Seip, doença rara que consiste em lipodistrofia generalizada congênita^20^. O medicamento, que ainda hoje não possui registro no Brasil, é um análogo recombinante do hormônio humano leptina e havia sido registrado no FDA em fevereiro de 2014 para a indicação clínica^21^. Cada ampola de metreleptina custava em torno de US$ 1.766,40^22^, com estimativa de gasto acima de quatro milhões de dólares por ano para tratar os pacientes. Naquele momento, os dados de eficácia e segurança do medicamento para a doença eram escassos, e na base de dados da EuroScan não havia estudo de MHT sobre o tema.

O relatório interno de MHT^23^ continha informações sobre a doença, os dados epidemiológicos do Brasil e do mundo, a descrição do medicamento, os custos estimados do tratamento com a metreleptina e as evidências de eficácia e segurança, além das situações regulatória e de pesquisa clínica para a indicação terapêutica. Apesar da evidência fraca a favor do uso do medicamento para o tratamento da doença, as decisões judiciais foram favoráveis ao seu custeio.

Posteriormente, o relatório de MHT^23^ foi compartilhado com a Argentina. A requisição por informações foi feita por meio da Rede de Avaliação de Tecnologias em Saúde das Américas (RedETSA). Na Argentina, o medicamento estava sendo requisitado para uso compassivo de pacientes com a doença, o que poderia resultar em gastos elevados, assim como no Brasil.

### Alertas

O objetivo dos alertas foi predizer o impacto de tecnologias novas e emergentes demandadas por via judicial, assim como daquelas que poderiam ser demandadas por essa via no futuro. Os destinatários das informações foram juízes, pacientes, profissionais de saúde e gestores, o que se refletiu na utilização de linguagem simples e extensão breve. Os alertas contemplaram uma tecnologia para uma indicação terapêutica^18^.

Produziram-se seis alertas no período: sofosbuvir associado a ledipasvir para Hepatite C crônica genótipo 1, tartarato de eliglustate para doença de Gaucher tipo 1, mipomersen para hipercolestorolemia familiar homozigótica, ivacaftor para fibrose cística, dispositivo médico para insuficiência mitral grave em pacientes de alto risco cirúrgico e aducanumabe para goença de Alzheimer (Tabela 1).

### Informes

Os informes tiveram o propósito de apresentar as potenciais tecnologias novas e emergentes para uma condição de saúde, contemplando várias tecnologias (Tabela 1). No período avaliado foram produzidos dois informes: “Stents bioabsorvíveis na intervenção coronária percutânea” e “Medicamentos em desenvolvimento para o tratamento da polineuropatia amiloidótica familiar associada à transtirretina”. Esses informes contemplaram duas categorias de tecnologias, dispositivos médicos e medicamentos (Figura 1), tendo como principal público-alvo os profissionais da saúde e a academia.

### Seções de MHT nos Relatórios de Recomendação

Os relatórios de recomendação da Conitec são documentos oficiais do MS que contemplam as evidências científicas, a avaliação econômica e a avaliação do impacto orçamentário das tecnologias em saúde submetidas à análise para incorporação no SUS^5^. As seções de MHT tiveram a finalidade de apresentar os medicamentos que pot encialmente poderiam competir com aquele que estava sendo analisado para incorporação no SUS, seja por nova via de administração ou por representar uma nova classe terapêutica, por exemplo, de maneira a apoiar as discussões da Conitec^5^.

Foram realizadas análises-piloto do horizonte tecnológico dos medicamentos em desenvolvimento clínico para a esclerose múltipla durante as avaliações das demandas de incorporação do fingolimode e da teriflunomida, em 2014 e 2016, respectivamente. Os achados do MHT foram apresentados ao plenário da Conitec, influenciando na recomendação emanada. Devido ao papel do MHT para a tomada de decisão, esse produto passou a ser realizado sistematicamente para cada medicamento em análise pela comissão^24^ a partir do ano de 2017.

Entre 2017 e julho de 2018, foram elaboradas 58 seções de MHT, perfazendo uma média de três seções por mês. Foram prospectadas 153 tecnologias novas e emergentes. Os temas de saúde mais frequentes foram doenças raras (23%), oncologia (19%) e neurologia (17%). Além de constarem no relatório de recomendação, as informações foram apresentadas oralmente ao plenário (Tabela 1) e resultaram em repercussões nas discussões e processos de tomada de decisão.

Uma dessas repercussões ocorreu no contexto da análise para incorporação dos medicamentos adalimumabe, etanercepte, infliximabe, secuquinumabe e ustequinumabe, para psoríase moderada a grave^25^. O MHT apontou 13 medicamentos para a indicação clínica, e os dados de eficácia do guselcumabe, do ixequizumabe e do brodalumabe sugeriam superioridade em relação ao adalimumabe, ao etanecerpte, ao infliximabe e ao ustequinumabe.

Além disso, três medicamentos emergentes (pliclidenoson, tofacitinibe e voclosporin) estavam sendo desenvolvidos para administração por via oral. Por outro lado, todos os medicamentos em análise para incorporação eram administrados por via subcutânea ou intravenosa, indicando uma potencial repercussão positiva dessas tecnologias na adesão terapêutica dos pacientes, caso fossem registradas no País.

O cenário de medicamentos potencialmente mais eficazes e com maior comodidade terapêutica para o paciente num horizonte próximo resultou na intensificação do debate no plenário da Conitec sobre recomendar preliminarmente a “não incorporação” de três dos medicamentos em análise.

No ano de 2017, o reconhecimento institucional do papel-chave do sistema de MHT da Conitec ao fornecer informações estratégicas sobre tecnologias novas e emergentes ao MS resultou na inclusão legal do monitoramento de tecnologias novas e emergentes como atividade do departamento assessor da comissão, como ferramenta para a redução das ações judiciais em saúde^26^.

## DISCUSSÃO

Este trabalho descreve os produtos de MHT desenvolvidos pelo MS do Brasil com vistas a subsidiar a avaliação de tecnologias em saúde no contexto de um dos maiores sistemas de saúde do mundo. Os resultados demonstram que o manual da EuroScan^7^ tem sido aplicável ao sistema de MHT brasileiro. O aumento do número de produtos elaborados por ano evidencia que, ao longo do tempo, o potencial do MHT como instrumento de coleta, síntese e apresentação de informações sobre tecnologias novas e emergentes foi sendo adotado nos processos de tomada de decisão no MS.

Demonstrou-se que, embora no âmbito de um mesmo sistema de MHT, as etapas do MHT não foram empregadas de forma única, de modo a construir diferentes produtos para fornecer informações para públicos específicos. O trabalho aponta um diferencial dos produtos de MHT brasileiros: sua estreita relação com a judicialização da saúde, em que tecnologias são requeridas por meio do poder judiciário. Isso pode ser explicado pelo fato de que esse mecanismo de acesso tem sido porta de entrada de medicamentos de alto custo com grande potencial de difusão inadequada e uso irracional, sobremaneira de tecnologias sem registro na Anvisa^27^. De acordo com Douw et al.^28^ (2003), os sistemas de MHT têm o propósito de auxiliar a racionalizar os processos de adoção e difusão de novas tecnologias. O potencial efeito positivo da ATS no preparo das defesas em casos de judicialização da saúde tem sido apontada^29^.

O MHT tem informado o sistema judiciário brasileiro por meio dos argumentos apresentados nas peças de defesa do MS em demandas judiciais e, secundariamente, por meio dos alertas e informes. Isso se deve ao fato de que esses produtos de MHT aparecem entre os resultados de buscas sobre tecnologias ainda sem registro na Anvisa em ferramentas como o Google®, uma vez que são disseminados por meio do sítio eletrônico da Conitec.

O compartilhamento do relatório interno da metreleptina com a Argentina, caso apresentado neste estudo, demonstra que os produtos de MHT da Conitec podem ser utilizados por outros países que lidam com a judicialização e também com outras finalidades. A geração de evidências científicas foi assinalada como uma das estratégias prioritárias para a abordagem da judicialização da saúde na América Latina e Caribe^30^, demonstrando o potencial de uso das informações providas pelos produtos de MHT para a região.

No trabalho de Packer et al.^9^ (2015) sobre a estrutura, processos e produtos de 15 membros da EuroScan, 80% dos sistemas de MHT estudados reportaram ter envolvimento de especialistas na revisão por pares. Parte dos produtos de MHT da Conitec também são revisados por pares. Assim como a maioria dos sistemas de MHT reportados no referido estudo, os dados deste trabalho apontam haver mais de um grupo de usuários potenciais das informações geradas por esse sistema.

Assim como os relatórios de MHT da agência sueca, os produtos brasileiros não constituem uma avaliação completa da tecnologia nova ou emergente, mas fornecem aos gestores informações precoces sobre elas^11^.

Da mesma forma que outros membros da EuroScan, a equipe de MHT do sistema da Conitec é pequena, composta por três pessoas envolvidas também com outras atividades. O sistema de MHT da Conitec está inserido em um departamento que atua em atividades de avaliação de tecnologias em saúde, não constituindo, portanto, uma instituição separada, também como outros membros da rede internacional^9^.

A literatura acerca dos resultados das atividades de MHT é escassa^9^^,^^32^^,^^33^. Foi possível demonstrar que as informações providas pelas seções de MHT nos relatórios de recomendação têm influenciado as recomendações da Conitec. Isso permite afirmar que o sistema descrito neste trabalho gera repercussões na tomada de decisão para a incorporação de tecnologias no SUS.

Sob essa perspectiva, o aumento do número produtos do tipo “seções de MHT nos relatórios de recomendação” nos anos de 2017 e 2018 demonstram sua relevância para o MS. Em 75% dos sistemas de MHT, o principal propósito das atividades é dar suporte à tomada de decisão quanto à cobertura e reembolso^9^, funções semelhantes às das seções de MHT nos relatórios de recomendação.

Um passo importante do sistema de MHT brasileiro será realizar estudos voltados para doenças específicas^31^ e entregar tais informações às áreas temáticas correspondentes do MS, a fim de indicar tecnologias com potencial de incorporação no SUS. No Reino Unido, as atividades de MHT fornecem ao *National Institute for Health and Care Excellence* (NICE) informações voltadas para a incorporação de tecnologias^32^.

Apesar de a Conitec não poder pautar proativamente as tecnologias que serão analisadas para incorporação, as áreas internas do MS podem demandar à comissão essas avaliações^4^. Assim, a consecução do passo discutido no parágrafo anterior poderá resultar num perfil mais proativo de atividades desempenhadas pelo sistema de MHT da Conitec. Nesse sentido, a expectativa de que o sistema de MHT fosse capaz de identificar tecnologias em fase inicial de adoção que precisam ser avaliadas para uso adequado tem sido em parte alcançada^13^.

A tradução dos informes e alertas para o inglês e espanhol e a disponibilização desse material na base de dados da EuroScan e no sítio eletrônico da Conitec permitirá o uso das informações por instituições que desenvolvem atividades de ATS e de MHT no mundo todo. Outros desafios para o sistema de MHT da Conitec serão apresentar informações a respeito da obsolescência e desabastecimento de medicamentos^12^, além de aperfeiçoar as estratégias de disseminação dos produtos desenvolvidos.

A construção do sistema de MHT brasileiro tem envolvido o Ministério da Saúde, a academia e outras partes interessadas. Essas atividades resultaram no reconhecimento institucional da metodologia como importante fase da ATS para informar os gestores de saúde com a melhor evidência disponível quanto a tecnologias novas e emergentes com potencial de provocar implicações legais, éticos, organizacionais e no cuidado oferecido aos pacientes.
